# Relationships between Motor Skills and Academic Achievement in School-Aged Children and Adolescents: A Systematic Review

**DOI:** 10.3390/children11030336

**Published:** 2024-03-12

**Authors:** Lijing Wang, Lijuan Wang

**Affiliations:** School of Physical Education, Shanghai University of Sport, Shanghai 200438, China; 2011111013@sus.edu.cn

**Keywords:** school children, motor skills, academic achievement, review

## Abstract

Purpose: This review systematically summarizes the studies of the relationship between primary-to-secondary school students’ motor skills and academic achievement, and analyzes the relationship between gross and fine motor skills and performance in different subjects. Method: Five electronic databases, Web Of Science, PubMed, PsycINFO, SPORTDiscus, and Academic Search Premier, were searched in March 2023. Semi-quantitative assessment methods were used to analyze the results of the included studies. Results: Seventy-eight articles were included in this systematic review. The semi-quantitative assessment results showed that gross (+, 65.0/62.5%) and fine motor skills (+, 83.3/80%) were positively correlated with overall performance and language performance, with ≥60% of the associations in the same direction. For different subjects, fine motor skills were positively correlated with students’ mathematics (+, 75.0%), reading (+, 72.7%), writing (+, 66.7%), and spelling (+, 60.0%) scores. However, the association between gross motor skills and students’ mathematics achievement (?, 52.8%), reading (?, 53.8%), and spelling (?, 50.0%) is uncertain, with <60% of the associations in the same direction. Conclusions: It is wise to direct our gaze toward the evolution of motor skills among students, especially primary school students. Different motor skill intervention modes should be selected in a targeted manner according to different subject achievements.

## 1. Introduction

Cognitive changes occurring in childhood and adolescence are critical to acquire the capability of learning and executing motor skills. The earlier cognitive theory suggested that motor and cognitive skills have been regarded as completely different processes which develop independently and involve diverse territories within the brain [1]. With the development of the cognitive psychology theory and the advancement of neuroscientific technical means, significant breakthroughs have been achieved in the study of motor skills and cognitive ability and the academic performance of children and adolescents, attracting widespread attention within the academic community [2].

According to the “embodied cognition” model presented by Piaget [3], the body and brain function as a single, interconnected unit within the “brain–body” system, with their operations being mutually dependent and closely interwoven [4,5]. Specifically, knowledge is constructed through motor behaviors which lead to the creation of mental representations [3] and promote the development of complex intellectual reasoning abilities [6]. Neuroscientific and psychological evidence suggests that motor skills allow students to understand better and develop more advanced cognitive abilities, such as reasoning, planning, and judgment, promoting academic performance [7,8]. Investigating the relationship between motor skills and cognitive function holds significance in attaining a thorough comprehension of changes occurring in children and adolescents.

The theory of cognitive load provides a new perspective. The theory postulates that the processing power for a task is limited [9]. The cognitive load theory posits that children possessing robust motor skills within the classroom environment are not compelled to allocate attentional focus, resources, or energetic exertion toward behavioral endeavors [10]. Children endowed with robust attentional stability, self-regulation, and operational memory may engage effortlessly in novel and intricate educational tasks [11]. These explanations emphasize the significance of examining the link between motor skills and academic achievements (AAs), as well as determining whether this association is limited to specific types of skills.

Motor skills encompass the learned sequences of movements that are combined to perform a task smoothly and efficiently [12]. In addition to movement, motor skills include the cognitive processes that produce movement [13]. Motor skills and cognitive abilities are mutually dependent, as they share common neural pathways within the brain. Functional neuroimaging has uncovered the profound activation of the neocerebellum and dorsolateral prefrontal cortex during the execution of numerous motor and cognitive undertakings [14,15]. Cognitive abilities exhibit a strong correlation with AA [16,17].

Motor skills are classified as gross motor skills (GMSs) and fine motor skills (FMSs). GMSs coordinate large muscle groups for the execution of basic movements such as sitting, crawling, walking, and running [18]. Fine motor skills (FMSs) involve coordinating small muscle movements for grasping, drawing, writing, and playing an instrument [11]. Multiple investigations have delved into the association between GMSs, FMSs, and AAs among children, yielding inconsistent results [19,20,21]. Some studies have indicated a positive correlation between GMSs [22,23,24] and FMSs [25,26,27], and AAs. Conversely, other research revealed no substantial linkage between these motor skills and students’ AAs [28,29,30]. By integrating the findings from these studies, we can comprehensively and objectively evaluate the associations between the two motor skill types and AAs among children.

Macdonald and collaborators’ study, which reviewed 55 studies from 2000 to 2018, examined the relationship between motor proficiency and mathematics and reading achievement in children aged 22 months to 18 years [31]. Their findings demonstrated a strong and positive correlation between fine motor integration, as well as an overall fine motor score, and performance in mathematics and reading (it was only found in some studies that looked at fine motor integration, while other studies looked at overall fine motor coordination). The research also showed a clear and positive association between speed, agility, coordination of the upper limbs, and the overall gross motor score on one hand, and mathematical and reading proficiency on the other. However, numerous works have explored the relationships between motor skills and AAs since 2018. These studies are important to further summarize the relationship between the two variables, and a comprehensive summation and analysis of these studies have yet to be undertaken. Moreover, studies that focused on the relationship between motor skills and different subjects, including language, writing, science, spelling, and overall academic achievement among children and adolescents, were not reviewed. Thus, to better understand the relationship between motor skills and AA, a more extensive review to summarize the achievement of different subjects is necessary. This review focused on school students’ academics (i.e., from primary to high school students). It aimed to (1) systematically summarize the studies on the relationships between school students’ motor skills and AA before 2023, and (2) quantify the association between motor skills and achievement in different subjects. This review can contribute to the development of interventions to increase motor skill participation and improve AA for school-aged students.

## 2. Methods

This review adhered to the Preferred Reporting Items for Systematic Reviews and Meta-Analyses Guideline (PRISMA) [32]. The protocol for this systematic review is registered on the International Platform of Registered Systematic Review and Meta-analysis Protocols database on 9 February 2024 (INPLASY202420043), and is available in full at inplasy.com (https://doi.org/10.37766/inplasy2024.2.0043, 9 February 2024). 

### 2.1. Search Strategy

Five electronic databases, Web Of Science, PubMed, PsycINFO, SPORTDiscus, and Academic Search Premier, were searched in March 2023. The search terms were based on the combination of three main areas: (1) student* OR youth* OR teenage* OR child* OR juvenile* OR adolescent*; (2) motor skill* OR motor competence OR motor ability* OR motor development OR motor coordination OR gross motor skill* OR fine motor skill*; and (3) academic achievement OR academic performance OR academic grades OR scholastic achievement. 

### 2.2. Inclusion and Exclusion Criteria

The inclusion criteria were as follows: (1) studies examining the associations between motor skills and AA were included, (2) studies that assessed gross or fine motor skills separately were included, (3) the participants were typically developing primary to high school students, (4) studies published in peer-reviewed journals in English until March 2023, and (5) quantitative studies. 

The exclusion criteria were as follows:(1) studies focusing other topics, (2) studies utilizing measurement batteries that incorporate fine motor skills as a component of the overall score, (3) people with disabilities, and people with diseases or disorders that affect or limit their physical activity, such as developmental coordination disorder (DCD) or autism spectrum disorder (ASD), (4) unpublished dissertations, conference proceedings, literature review, and comments were excluded, and (5) qualitative studies, case reports, and expert opinions were disregarded. The two authors assessed the identified studies independently based on the inclusion and exclusion criteria. The second author judged on the disagreement. 

### 2.3. Quality Assessment

The researchers used the adapted McMaster Critical Review Form Quantitative Studies to determine the methodological quality [33]. The form was selected due to its demonstration of a strong inter-rater agreement rate, ranging from 75 to 86% [34]. It encompasses 16 items, addressing various aspects of the study such as its intent, context, project, sampling, measurements, data analysis techniques, conclusions, and implications, as well as limitations. The researchers assigned scores to all items based on the fulfillment of specific criteria, awarding ‘yes’ a score of 1 and ‘no’ a score of 0. Two reviewers independently assessed all studies. The final score for each study was calculated by summing the total scores of the relevant items and dividing it by the total scores. The second author resolved any uncertainty and disagreement. Scores falling within the ranges of less than or equal to 50%, between 51 and 75%, and greater than 75% were categorized as indicating low, good, and excellent quality, respectively.

### 2.4. Data Extraction and Synthesis

The first author performed the data extraction, and the second author performed the final validation. An overview of the demographic characteristics of the participants across these studies were conducted and included the type of research, participant characteristics, motor skill measurement methods, and methodological quality. The participants were divided into four groups. The methodological quality was graded according to three levels: high, moderate, and low (Table 1).

### 2.5. Coding of Analysis

A combined qualitative–quantitative analysis was employed to gauge the congruency among distinct relationship varieties [35]. This assessment is presented in the report with descriptive results and also provides additional objective evidence [36]. The identified association for every result in each study was marked as positive or negative. The code “0” indicates a non-significant association between motor skills and different subject achievement. The codes “+” and “−” indicate whether the association between motor skills and various subject achievement is positive or negative, respectively. When findings were reported separately according to sex, these were noted as “male” or “female”. Subsequently, we delved deeper into coding these relationships by meticulously analyzing the studies that underpin the correlations. Relationships observed only once remained unelaborated in the text and were subsequently coded as “No Description (ND)” [12]. When variables were encountered two or more times, we strictly followed the method framework established by Sallis and carried out systematic sorting [35]: (1) “+” or “−”: positive or negative correlation when the variable appeared in two or more studies, with ≥60% of the associations in the same direction; (2) “?”: inconsistent or uncertain correlation, with 34–59% of the associations in the same direction; (3) “0”: no association, with 0–33% of the associations in the same direction. 

**Table 1:** Descriptive statistics for studies used in the systematic review.

Description	*n* (%)
Type of research	
Longitudinal studies	35 (44.9)
Cross-sectional studies	31 (39.7)
Experimental studies	12 (15.4)
Sample size	
<100	17 (21.8)
100–249	22 (28.2)
250–500	16 (20.5)
>500	23 (29.5)
Motor skill assessment	
MABC/MABC-2 [37,38]	12 (15.4)
BOT-2 [39]	7 (9.0)
VMI [40]	7 (9.0)
KTK [41]	5 (6.4)
Early Screening Inventory [42]	4 (5.1)
Methodological quality	
Excellent	55 (70.5)
Good	18 (23.1)
Low	5 (6.4)

Note: MABC/MABC-2: Movement Assessment Battery for Children-2; BOT-2: Bruininks–Oseretsky Test for Motor Proficiency; VMI: Visual Motor Integration test; KTK: Körperkoordinationtest für Kinder. Quality evaluation details are listed in Supplementary Material.

## 3. Results 

### 3.1. Search Results 

**Figure 1:**
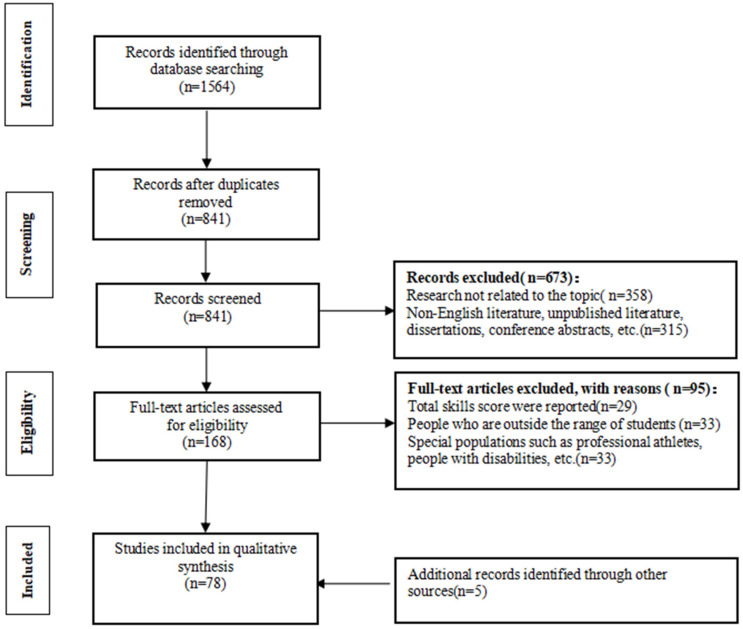
Flow diagram of the literature search.

The four-phase methodology used to identify, evaluate, and ultimately choose studies for this extensive review is concisely summarized in the PRISMA diagram [32] (Figure 1). The initial search identified 1564 studies. Duplicates among the studies were removed upon exporting them to the EndNote X2 software. Subsequently, the screening of the remaining 841 articles was conducted by examining their title and abstracts, leading to the exclusion of 673 studies. The remaining 168 studies were re-screened by reading the full text; 73 were initially identified. Five relevant studies were added by reading the literature and references. The ultimate count of studies incorporated into the systematic review stood at 78 (see Figure 1).

### 3.2. Methodological Quality

Out of the 78 studies included in the review, 55 (70.5%) were deemed to have excellent methodological quality, 18 (23.1%) were categorized as good quality, while 5 studies fell into the low-quality category. Notably, the weakest aspect among the included studies pertained to sampling techniques and procedures.

### 3.3. Study Characteristics

The studies encompassed in this review were published between the years 2001 and 2022. Most of these studies were longitudinal studies (*n* = 35, 44.9%), and the remaining were cross-sectional (*n* = 31, 39.7%) and experimental studies (*n* = 12, 15.4%). The total samples in each study included in the review ranged from 13 to 34,491. Most studies (*n* = 55, 70.5%) had <500 samples. The research focused on primary to high school students (aged 4–17), and most studies (*n* = 72, 92.3%) focused on primary students. Motor skills were assessed by using a range of instruments such as the Movement Assessment Battery for Children-2 (MABC/MABC-2) (*n* = 12, 15.4%), Bruininks–Oseretsky Test for Motor Proficiency (BOT-2) (*n* = 7, 9.0%), the visual motor integration test (VMI) (*n* = 7, 9.0%), Körperkoordinationtest für Kinder (KTK) (*n* = 5, 6.4%), Early Screening Inventory (*n* = 4, 5.1%), and other Early Screening Tools (Table 1). 

### 3.4. Relationships between GMSs and AA

Table 2 presents findings regarding the association between GMSs/FMSs and AA among students. A cumulative total of 42 studies delved into the association between gross motor skills and academic performance. The relationships between GMSs and overall performance (i.e., composite scores and GPA) and AA in mathematics, reading, writing, spelling, and language were studied twice or more. GMSs are consistently and positively related to the overall academic and language performance, with 65.0% and 62.5% of the studies supporting this view. GMSs were positively associated with mathematics, reading, and spelling performance, with only 52.8%, 53.8%, and 50% (less than 60%) of the studies supporting this view, thus making the association uncertain. No correlation was found between GMSs and students’ writing skills because only 28.6% of the studies supported the relationship. 

### 3.5. Relationships between FMSs and AA

A total of 57 studies examined the relationship between FMSs and AA. The relationships between FMSs and overall performance and AA in mathematics, reading, writing, spelling and language were studied twice or more. FMSs demonstrate a consistent and positive correlation with overall academic achievement and mathematics, reading, writing, spelling and language performance, with 83.3%, 75.0%, 72.7%, 66.7%, 60.0%, and 80.0% of the studies supporting it, respectively.

## 4. Discussion

This review offers a comprehensive and systematic compilation of studies examining the relationship between motor skills and AA among school-aged students. Findings showed that GMSs and FMSs are consistently and positively related to the overall academic performance. 

The “embodied cognition” model may explain why GMSs and FMSs are positively associated with the overall AA. Moreover, the results showed that GMSs and FMSs are consistently and positively associated with students’ language performance. Developing motor skills and sensorimotor experience can be conductive to acquire some linguistic categories, including verbs or spatial vocabulary (e.g., locative adverbs and prepositions, and verbs indicating movements in a direction) [109]. The Piagetian constructivism perspective is that children learn and create by actively interacting with their environment through gross motor (e.g., walking) and fine motor (e.g., grasping) activities [110]. Their sensorimotor experiences can also build their vocabulary and linguistic skills. Many neuroimaging studies have shown that brain activations take place in the motor and sensory cortices that correspond to the communicated significance when language is processed [111]. A handful of experimental studies have corroborated that the controlled practice of motor skills, both gross and fine, during the language acquisition process facilitates the transformation of abstract information into tangible and concrete concepts, ultimately leading to optimal learning outcomes [6,112,113].

Reading, spelling, and writing are different subjects related to FMS. Reading requires FMSs controlling eye movement for word tracking. Speaking and spelling require FMSs that control the production of sound and written content. The process of writing work demands precision in hand movements and the synchronization of the hand and eye [76]. Fine motor writing skills play an important role in creating student learning conditions. In their review of studies examining embodiment across various cognitive domains, Kiefer and Trump demonstrate that the adoption of writing techniques can influence reading performance, as they stimulate motor programs and evoke sensory experiences linked to writing while engaging in the reading task [114]. Therefore, individuals who engage in handwriting exhibit superior letter recognition during reading tests compared to those who rely on typewriting. This observation underscores the argument that deliberate sensory–motor experiences contribute to the development of robust sensory–motor memory traces, subsequently bolstering the efficiency of learning. 

Some research shows that students can learn to spell words more accurately through handwriting [97,115,116]. First, handwriting tasks allow children to establish intrinsic structures for the symbolic systems underlying successful academic performance, serving as the fundamental building blocks for various academic disciplines [100]. Second, research indicates that when students are able to utilize handwriting to record their thoughts and ideas effortlessly, they conserve cognitive resources, enabling them to produce more intricate compositions [117,118]. Handwriting fluency enables students to focus all their energy on creating content, without having to split their attention between content development and text production [116].

The review also found that FMSs can improve mathematics performance, consistent with the results summarized by Macdonald (2018) [31]. In this review, most studies (92%) focused on Grades 1 to 6 students, and only one focuses on secondary school students. The correlation between FMSs and mathematics performance is reflected mainly during primary schooling. Understanding mathematics is grounded in our bodily interactions and experiences with the physical world [119]. Hill [120] (2010) contended that the acquisition of motor skills, which encompasses the refinement of physical control, is intricately linked to cognitive performance and academic achievement. This places an emphasis on the significance of skills in the realm of educational pursuits. Primary school mathematics classes use hands to operate toys, cards, and other methods to help students master the basic concepts of numbers or some symbols (e.g., one-to-one correspondence, counting, sorting) to cultivate and improve their computing ability [88]. The “neurological” perspective suggests a close proximity between the brain regions responsible for finger movement and those underlying arithmetical proficiency [121,122]. Consequently, practicing FMSs is believed to stimulate these brain regions, subsequently enhancing mathematical performance. 

Furthermore, numerous physical games that demand motor skills possess the capability to foster and enhance the development of mathematical abilities and concepts, encompassing spatial awareness, classification, counting or sorting, as well as directional and distance comprehension. Early motor skill proficiency has been found to influence the subsequent development of cognitive skills, and children with better motor development tend to exhibit better cognitive and academic outcomes in adolescence [48,123].

Although a preliminary discussion of the relationship between GMSs and mathematics, reading, and spelling performance was conducted, the research results are uncertain, and such a relationship needs to be further explored and clarified.

## 5. Limitations and Implications for Future Research 

This research, however, is not without its limitations. First, only studies published in English were considered, potentially excluding some pertinent research. Second, due to the heterogeneity among studies, a meta-analysis was not feasible. Moreover, a limited number of studies have accounted for the potential impact of additional variables, including demographic characteristics, socioeconomic status, body mass index, and ethnic background. Therefore, we only examined the relationship between motor skills and AA. 

Despite the limitations, the present review’s findings offer valuable insights into potential future research directions and practical applications. First, researchers should consider using standardized instruments to assess motor skills and academic performance to reduce heterogeneity between studies and more accurately compare the results of different studies. Second, further attention should be given to the scores of the subjects not supported by sufficient evidence (such as history and science) and subjects that do not draw definite conclusions due to contradictory evidence (such as reading), and the relationship between motor skills and these subjects clarified. Third, research in the future should pay more attention to mediating variables and exploring more mediating variables from physiology and cognitive psychology perspectives to understand the internal mechanism of the motor skills affecting academic performance, this will be fraught with challenges. Finally, the relationship between motor skills and academic achievement may not apply to populations (e.g., migrants and immigrants, children from high socioeconomic status backgrounds, children with low IQs, etc.). Hence, it is imperative to delve deeper into the disparities in the association between motor skills and AA among students of different characteristics, including gender, age, intelligence level and family background, which must be thoroughly studied. 

The research findings offer valuable insights for educational practitioners. Understanding the importance of motor skills in children is crucial for educators, as they play a pivotal role in enhancing academic performance and fostering progress in various domains. PE teachers should give due diligence to the development of motor skills and actively develop some motor skill games suitable for different ages so as to promote physical progress and AA. Cognitive and educational psychologists need to explore the relationships and mechanisms between different skills and AA more actively in order to facilitate innovation within school welfare policy formulation and teaching methods. 

## 6. Conclusions

To sum up, using a semi-quantitative evaluation of the research results, this systematic review revealed a positive correlation between motor skills and certain subdomains of AA. There is some experimental research suggesting that implementing motor skill interventions with school settings may have a positive impact on AA, especially in math and reading. 

Hence, future interventions should focus on promoting motor skills, which is one of the most comprehensive and effective approaches to promoting AA. Public policies may need to be changed to systematically provide incentives and guidance for improving school sports skills. At the same time, more research is required that employs cutting-edge technology, such as functional magnetic resonance imaging (fMRI) and EEG (electroencephalogram), to elucidate the biological underpinnings that underlie the observed impact on cognitive faculties and AA. Furthermore, prolonged randomized controlled trials (RCTs) are imperative to establish a causal link between enhanced motor proficiency and the improvement in academic outcomes.

## Figures and Tables

**Table 2:** Summary of studies on the relationships between GMSs/FMSs and AA.

Summary Coding		Associated		Not Associated		Summary Coding		
Motor Skill	Subject	References No.	(−/+)	References No.	0	*n/N*	(%)	Coding
GMS	Math	[22,23], [43] ^F^, [43] ^M^, [44] ^F^, [44] ^M^, [45,46,47,48,49,50,51,52], [**53**,**54**,**55**,**56**,**57**]	+	[10,19,29], [30] ^F^, [30] ^M^, [43] ^F^, [47,50,58,59,60,61,62,63,64,65], [**66**]	0	19/36	52.8	?
	Reading	[19,23], [44] ^F^, [46,48,49,50,51,52,67], [**55**,**56**,**68**,**69**]	+	[10,29], [30] ^M^, [30] ^F^, [44] ^M^, [49,50,59,63,64,65], [**57**]	0	14/26	53.8	?
	Writing	[19], [30] ^M^	+	[29], [30] ^F^, [63,64], [**56**]	0	2/7	28.6	0
	Spelling	[**57**,**69**]	+	[23,50]	0	2/4	50.0	?
	Language	[43] ^F^, [43] ^M^, [44] ^F^, [46], [**54**]	+	[58,60]	0	5/8	62.5	+
		[44] ^M^	−					
	History	[43] ^F^, [43] ^M^	+	[43] ^F^, [43] ^M^	0			ND
	Overall Performance	[19,24], [43] ^F^, [43] ^M^, [44] ^F^, [70,71,72,73,74,75], [76] ^F^, [**77**]	+	[10], [44] ^M^, [58,64,72,76] ^M^	0	13/20	65.0	+
		[78]	−					
FMS	Math	[10,16,20,26,48,49,51,52,59,60,61,62,63,64,65,79,80,81,82,83,84,85,86,87,88,89,90,91,92,93,94], [**53**,**95**]	+	[19,29,49,50,59,60,64,83,88,91,96]	0	33/44	75.0	+
	Reading	[10,25,28,48,49,52,59,63,64,65,67,80,82,83,84,85,86,89,90,93,94,96,97,98,99,100,101,102,103,104], [**68**,**105**]	+	[19,25,28,49,50,51,59,67,84,89,91], [**105**]	0	32/44	72.7	+
	Writing	[63,64,83,96,104], [**106**]	+	[19,29], [**106**]	0	6/9	66.7	+
	Spelling	[25,94,100]		[25,50]		3/5	60.0	+
	Language	[26,60,81,96]	+	[60]		4/5	80.0	+
	Science	[26]	+					ND
	Overall Performance	[10,26,64,75,78], [76] ^F^, [83,100,107,108]	+	[19], [76] ^M^	0	10/12	83.3	+

Note: (1) “No Description (ND)”: when the number of studies is 1; “+” or “−”: positive or negative correlation when the variable appeared in two or more studies, with ≥60% of the associations in the same direction; “?”: inconsistent or uncertain correlation, with 34–59% of the associations in the same direction; “0”: no association, with 0–33% of the associations in the same direction. (2) Four different studies investigated the correlation between motor skills and academic performance in boys and girls, and the findings were reported separately by gender. (3) Bold serial numbers are intervention studies. ^M^: for male; ^F^: for female.

## Data Availability

The data presented in this study are available in Supplementary Materials.

## Supplementary Materials

The following supporting information can be downloaded at: https://www.mdpi.com/article/10.3390/children11030336/s1, Table S1: Key data extracted from studies examining the relationship between motor skills and academic performance in school-aged children and adolescents; Table S2: Results of the study quality evaluation using the adapted McMaster Critical Review Form for Quantitative Studies.
