# Supplementary material for: Relationships between Motor Skills and Academic Achievement in School-Aged Children and Adolescents: A Systematic Review
Source: Children (Basel). 2024 Mar 12;11(3):336. doi: 10.3390/children11030336 (PMC10969452; doi:10.3390/children11030336)
Supplement: Supplementary file 1 [file children-11-00336-s001.zip › children-2862683-supplementary.pdf]

Table S1. Key data extracted.

References No.	Author、Year	Geographic Location#	Research Design	Sample Characteristics	Motor skill Measures		Academic performance Measures		Major Findings	Quality
					Content	Tools	Content	Method		
[10]	Pagani, 2010	Canada	Longitudinal Study (3 years)	65 months n=1155	GMS FMS	Coordinated; climb stairs; and overall physical development; (holding a pen, crayons, or a brush; and manipulate objects	Math、reading、general academic achievement	Teachers provided overall estimate	GMS were not correlation with math, reading, general academic achievement. FMS in kindergarten yielded significant results, predicting second grade reading, math, and general achievement (r=.11-.17,p <.01)	87.5% Excellent
[16]	Oberer, 2018	Switzerland	longitudinal study (18 months)	M = 6.42±0.32 n = 134	FMS	M-ABC-2	Math、reading	"Heidelberger Rechentest" (HRT 1–4); "Salzburger Lese-Screening" (SLS	Visual-Motor Coordination were significantly and positively correlated with second grade math and reading(r=.17-.52,p < .05)	87.5% Excellent
[19]	Pacheco, 2016	Brazil	Cross-sectional	8~11 years n=100	GMS FMS	BOT-2	Writing、Reading、Math and total scores	Standardized Academic Achievement Tests	Significant positive correlations between GMS and Writing, Reading scores and total score(OR=1.38，95%CI：1.0-1.26*；OR=1.19，95%CI=1.0-1.41*；OR=1.12，95%CI：1.0–1.21**）, no correlation with Math scores；FMS does not correlate with Writing, Reading and	87.5% Excellent

									Math scores and total scores	
[20]	Kim, 2017	USA	longitudinal study (2 years)	6.0-7.9 years n=119	FMS	Neuro-psychological assessment battery	Math	Key Math-3 Diagnostic Assessment	Fine motor coordination in first grade were no correlation with math in grade 2；Visuo-motor integration in the kindergarten were significantly and positively correlated with math scores in grade 1/2(β= .13-.14 ,p<0.001).	81.3% Excellent
[22]	Syväoja. 2021	Finland	Cross-sectional	12~17 years n=311	GMS	5-leaps test\ throwing–catching combination test	Math	Standardized Academic Achievement Tests	GMS are significantly and positively correlated with Math scores(b=0.04-0.13, p< .05)	81.3% Excellent
[23]	Bruijn, 2019	Netherlands	Cross-sectional	9.17 years n=891	GMS	KTK BOT-2	Reading、Math、Spelling	Standardized Academic Achievement Tests	GMS were significantly and positively correlated with Math scores(r=0.19,p< .01), Reading scores(r=0.10,p< .05), and No correlation with Spelling scores.	93.8% Excellent
[24]	Schmidt, 2017	Switzerland	Cross-sectional	10~12 years n=236	GMS	N	Math、Reading、Spelling total scores	Standardized Academic Achievement Tests	GMS were significantly and positively correlated with total academic achievement scores(β=0.47,p< .01).	81.3% Excellent
[25]	Lê, 2021	France	Cross-sectional	8.5 years n=278	FMS	MABC	Spelling and reading	N	Bimanual coordination are significantly and positively correlated with reading accuracy(r=.16, p < .05), manual dexterity are not correlated with	81.3% Excellent

									reading accuracy.<br>Manual dexterity are significantly and positively correlated with spelling(r=.17, p < .05), bimanual coordination are not correlated with spelling.	
[26]	Coetzee, 2020	South Africa	Cross-sectional	9.9 years n=863	FMS	VMI-4	Math、Language、Science and total scores	Standardized Academic Achievement Tests	FMS are significantly and positively correlated with Math, Language, Science scores and total academic performance(r=0.1—0.24,P < 0.05）.	81.3% Excellent
[28]	Milne, 2018	Australia	Cross-sectional	6.07 years n=24	FMS	BOT-2	Reading	Teacher Assessment	Fine Manual Coordination was correlation with Reading scores(r=0.44, p=0.04).<br>Fine Manual Control was not correlation with Reading scores.<br>Body Coordination Sentence was correlation with Sense Accuracy(r=0.45, P=0.03), not correlation with Reading Decoding.	93.8% Excellent
[29]	Costa, 2019	Brazil	Cross-sectional	10 years n=31	GMS FMS	MABC-2	Writing、Math、Reading	Standardized Academic Achievement Tests	No correlation between GMS and FMS and Writing, Math and Reading scores	87.5% Excellent
[30]	Chagas, 2016	Brazil	Cross-sectional	12~14 years n=122	GMS	KTK	Writing、Reading、Math	Standardized Academic Achievement Tests	GMS were significantly associated with Writing scores for boys(r=.284,p< .05).<br>GMS were not significantly associated with Reading and Math scores for both boys and girls, and Writing scores for girls	50% Low
[43]	Jaakkola, 2015	Finland	Longitudinal（2years）	13 years n=325	GMS	N<br>8 dribble test	Language、Math、History	Standardized Academic Achievement	For girls，GMS were significantly and positively correlated with Language(r=0.18,p< .01) and History scores(r=0.17,p< .05) and total	87.5% Excellent

								Tests	scores(r=0.16,p< .05) , but not with Math scores in Grade 7; GMS were significantly and positively correlated with Language and Math scores and total scores, but not with History scores in Grade 9.<br>For boys,GMS were significantly and positively correlated with Language(r=0.18,,p< .05), Math scores (r=0.18,p< .05)and total scores(r=0.19,p< .05), but not with History scores in grade 7. GMS were significantly and positively correlated with Language and Math、History scores and total scores in Grade 9.	
[44]	Aadland, 2017	Norway	Cross-sectional	10 years n=697	GMS	MABC-2	Language、Reading、Math and (total scores)	Standardized Academic Achievement Tests	For girls, a significant positive correlation between GMS and performance in Language, Reading and Math and composite score of academic performance(β=0.14–0.22,p< .05)；<br>For boys' GMS were significantly positively correlated with Math(β=0.17,p< .05) scores, significantly negative with Language scores(β= -0.01,p< .05), and not correlated with Reading scores and composite score of academic performance .	87.5% Excellent
[70]	Syvaoja, 2017	Finland	Longitudinal（2years）	9~15 years n=970	GMS	N	language、math, physics,	Standardized Academic Achievement	Better GMS at T2 predicted better total academic scores at T3 (B = 0.06, 99% CI = 0.00–0.11, Fig. 2A; B	87.5% Excellent

							chemistry, biology, geography, history, and religion or ethics (total scores)	Tests	= 0.06,99% CI = 0.01–0.11, Fig. 2B). GMS at T1 did not predict total academic achievement scores at T2.)	
[71]	Batez, 2021	Serbia	Cross-sectional	8.60 years n=130	GMS	KTK	Language、Math (total scores)	Standardized Academic Achievement Tests	GMS were significantly and positively correlated with total academic achievement scores(r=0.22,p< .05).	93.8% Excellent
[58]	Waal, 2020	South Africa	Longitudinal（7 years）	6.86 years n=816	GMS	BOT-2	Math、Language (total scores)	Teacher Assessment	GMS are not significantly and positively correlated with Math and Language scores and total academic achievement scores.	87.5% Excellent
[45]	Syväoja, 2019	Finland	Longitudinal（2 years）	12.5 years n=954	GMS	5-leaps test\ throwing–catching combination test	language、math、physics, Chemistry、biology、geography、history, and religion or ethics	GPA	GMS in year 2 predicted better GPA a year later (B = 0.06, 99% CI = 0.00–0.11; B = 0.06, 99% CI = 0.01–0.11)	81.3% Excellent

							(total scores)			
[46]	Aadland, 2017	Norway	longitudinal study (7- months)	10.2 ± 0.3 years n=1129	GMS	M-ABC-2	Math、reading、language	specific standardized Norwegian National tests	GMS was associated with mathematics, reading，language (r = 0.05-0.20, p =< .05).	81.3% Excellent
[47]	Lonneman, 2011	Germany	Cross-sectional	M=9.0 years，n=53	GMS	Static balance tasks	math	DEMAT 2+ German scholastic achievement test for mathematics	Significant partial correlations between GMS and multiplication (r=0.30-0.34, p<0.05); subtraction with borrowing (r=0.26-0.27, p<0.05) but not for less complex addition/subtraction tasks	75.0% Good
[72]	Lima, 2020	Denmark	longitudinal study (1 years)	M=7.87±0.34 years n=1020	GMS	KTK、(eye-hand-coordination—perceptuomotor integration	language、math (total scores)	standardized Danish language and math tests	Balance was longitudinally associated with academic performance(β= 0.126, 95% CI: 0.074 to 0.179)；Precision throw did not present total or mediated association with academic performance	68.8% Good
[73]	Lopes, 2013	Portugal	Cross-sectional	9-12 years n= 596	GMS	KTK	Language、Math (total scores)	National Exams	Children with normal coordination exhibited a higher probability of having high AA, compared with those with insufficiency GMS (p < .05)	93.8% Excellent
[74]	Fernandez-Sanchez, 2022	Spain	Cross-sectional	8-10 years n=451	GMS	MABC-2	language、math (total scores)	school's administration to determine	The direct path from GMC to AA was significant (b = 0.22, p = 0.022)	68.8% Good

[48]	Geertsen, 2016	Denmark	Cross-sectional	8~10 years n=423	GMS FMS	N	Math、Reading	Standardized Academic Achievement Tests	GMS and FMS were significantly and positive associated with Math scores(-0.22±0.03***）and Reading scores(-0.32±0.05***).<br>(i.e. the slower the time taken to complete fine and gross motor tasks, the lower the score on the maths skills test)	87.5% Excellent
[49]	Haapala, 2014	Finland	Longitudinal（3 years）	6-8 years n=174	GMS FMS	Balance Test、BBT	Reading、Math	Standardized Academic Achievement Tests	GMS in grade 1 are significantly and positively correlated with Reading Fluency(r = 0.16, p < 0.05)and math(r = 0.17, p < 0.05) in grade 3 , No correlation with reading Comprehension.<br>FMS in grade 1 were no correlation with reading and math in grade 3,were significantly and positively correlated with reading scores in grade 1 and 2(r = 0.18-0.20, p < 0.05) and math scores in grade 2(r = 0.18, p < 0.05) .	93.8% Excellent
[59]	Macdonald, 2020	Australia	Cross-sectional	6.77 years n=55	GMS FMS	BOT-2	Math、Reading	WIAT	No correlation between GMSs and Math and Reading scores.<br>Manual dexterity were not correlated with math and reading.<br>Fine motor integration were significantly and positively correlated with math(r = .525, p<.001) and reading(r = .470, p<.001)	87.5% Excellent

[50]	Rigoli, 2012	Australia	Cross-sectional	12~16 years n=93	GMS FMS	MABC-2	Reading、Math、Spelling	WIAT	Significant weak positive associations between GMS(aiming & catching) and word reading (r=0.280, p<0.01) and numerical operations (r=0.229, p<0.05), not correlated with Spelling scores.<br><br>Non-significant findings for FMS(manual dexterity) and GMS(balance) with word reading、numerical、spelling.	87.5% Excellent
[60]	Niekerk, 2015	South Africa	Cross-sectional	13~14 years n=236	GMS FMS	BOT-2	Language、Math	Final scores	GMS(Standing on one leg) was not significant positive correlations with math and language. FMS(fold paper on line)was Significant very weak-to-weak positive correlations between maths and language (r=0.13-0.15, p<0.05). FMS(Copy a star)not correlated with maths and language.	50.0% Low

[75]	Katagiri, 2021	Japan	Longitudinal （7years）	5~13 years n=2501	GMS FMS	Child Development Assessment Scales	Language 、Math (total scores)	Standardized Academic Achievement Tests	GMS were significantly and positively correlated with total achievement scores(r=0.17-0.19,p<0.01). FMS were significantly and positively correlated with total score of achievement(r=0.26-0.34,p<0.01).	87.5% Excellent
[61]	Fernández-Méndez. 2020	Spain	Cross-sectional	6~8 years n=305	GMS FMS	MABC-2	Math	Standardized Math Achievement Tests	GMS were not correlated with Math scores. FMS were significantly and positively correlated with Math scores(r=0.30,p<0.05）.	81.3% Excellent
[78]	Ricciardi , 2021	USA	Longitudinal Study (5 years)	4 years n=33717	GMS FMS	The Learning Accomplishment Profile Diagnostic (LAP-D)	(GPA)	Teacher-assigned grades/grade point average (GPA)	GMS in kindergarten were significant and negativity with 5th Grade GPA(-.043，P < 0.001） FMS in kindergarten were significantly and positively correlated with 5th Grade GPA(r=0.09, P < 0.001);	87.5% Excellent
[62]	Gandhi, 2012	Malawi	Prospective cohort study (12-years)	5 years n=415	GMS FMS	Developmental Assessment	Math	Math assessments	Non-significant associations found for GMS (T0) and mathematics (T1) (regression coefficient=0.206, p=0.176) (observed data); (regression coefficient=0.184, p=0.216) (imputed data) FMS (T0) was independently associated with mathematics score (T1) (regression coefficient=0.412, p=0.032) (observed data); (regression coefficient=0.445, p=0.011) (imputed data)	87.5% Excellent
[51]	Kurdek , 2001	USA	Longitudinal (4-5-years)	n=281	GMS FMS	Kindergarten Diagnostic	Reading、math	Ohio proficiency-based assessments	Significant positive associations between GMS (T0) and reading (T1) (r=0.17, p<0.01) and maths (T1) (r=0.17, p<0.01),	75.0% Good

						Instrument:			Significant weak positive association between FMS (visual motor integration) (T0) and maths (T1) (r=0.21, p<0.01).Non-significant associations between reading (T1)<br>Significant associations between the GMS and reading performance (r=-0.0117--0.251， p<0.05).	
[67]	Papadimitiriou, 2014	Greece	Longitudinal<br>(2-years)	M=5.6±0.36 years<br>n=300	GMS<br>FMS	Bead threading task;<br>shape copying task;<br>postural stability task	reading	Standardized Greek reading tests:	Significant positive associations between FMS (shape copying) (T0) and grade 1 reading performance (T1) (r=0.245, p<0.01), grade 2 reading performance (T2) (r=0.232, p<0.01).<br>Non-significant findings between FMS (bead threading) and reading performance in grades 1 and 2.	75.0%<br>Good
[52]	Son and Meisels,<br>2006	USA	Longitudinal<br>(18-months)	M=65±4.07 months;<br>n=12583<br>Kindergarten (T0)<br>Grade 1 (T1)	GMS<br>FMS	Early Screening Inventory-Revised:	Reading、math	early school achievement battery	Significant positive associations between GMS (T0) and reading (T1) (r=0.19, p<0.001) and maths (T1) (r=0.22, p<0.001)<br>Significant positive associations between FMS(visual motor skills) (T0) and reading (T1) (r=0.40, p<0.001) and maths achievement (T1) (r=0.48, p<0.001)	93.8%<br>Excellent
[63]	Giles,<br>2018	UK	Cross-sectional	5–11 years<br>n= 368	GMS<br>FMS	Balance tests , Kinematic Assessment Tool	Math、reading and writing	Nationally standardized academic-attainment scores	GMS showed no clear relationship with mathematical- or reading and writing scores.<br>FMS had a general significantly and positively relationship with mathematics, reading, and writing (data not shown)	75.0%<br>Good

[64]	Escolano-Pérez, 2020	Spain	longitudinal study (1 years)	M=5.72±0.30 years n=38	GMS FMS	Systematic observation Fine Motor Coordination: Fine Motor Coordination:	Reading、writing and Math、and total scores	standardized subtests	The GMS was not corelated with Reading、writing、Math and overall academic scores . FMS (Coordination and Integration) were associated with Reading and writing(β= 0.344, p = 0.025; β= 0.349, p = 0.024, respectively) and overall academic competency(β= 0.267, p = 0.065; β = 0.493, p = 0.001, respectively). Integration was associated with mathematics competency(β = 0.476, p = 0.002),Coordination was not associated with mathematics.	93.8% Excellent
[65]	Grissmer, 2010	USA	Longitudinal (5 years)	5-6 years n=1756	GMS FMS	ECLS-K study: Early Screening Inventory:	Reading、math	Achievement tests	GMS was not a significant predictor for mathematics achievement and reading achievement. Significant positive association found for FMS and reading achievement (β=0.07, p<0.00001) and maths achievement (β=0.14, p<0.00001) in ECLS-K study	68.8% Good
[76]	Ryu, 2021	Korea	Cross-sectional	fourth graders n= 110	GMS FMS	Soda pop hand test and soda pop foot test	Math、English、science (total scores)	final scores at the end of that semester	Girl's FMS(r=0.413, P < 0.05）and GMS (r=0.409，P < 0.05) were significantly and positively correlated with total Grades； Boy's FMS and GMS were No correlation with with total Grades.	75.0% Good
[107]	Fernandes, 2016	Brazil	Cross-sectional	8-14 years n=45	FMS	TTD	Writing、Reading、Math (total scores)	Standardized Academic Achievement Tests	FMS were significantly positively correlated with total academic achievement scores(r=-0.536,P < 0.01）.	81.3% Excellent

[79]	Duran, 2018	USA	Longitudinal （1years）	6.6 years n=73	FMS	NEPSY	Math	Math Achievement Tests	FMS are significantly and positively correlated with Math scores(β=0.26, P < 0.01）.	93.8% Excellent
[97]	Suggate, 2019	Germany	Longitudinal Study (9.47 months)	6 years n=120	FMS	M-ABC	reading	ELFE subtests	FMS in kindergarten were significantly and positively correlated with Grade 1 reading(r=0.53, P < 0.05)	75.0% Good
[80]	Sortor, 2003	USA	Cross-sectiona l	7~10 years n=155	FMS	VMI-4	Math、Reading	Standardized Academic Achievement Tests	FMS are significantly and positively correlated with Math scores(r= 0.274,P < 0.01) and Reading scores(r=0.163,P < 0.05）.	68.8% Good
[81]	Sulik, 2018	USA	Longitudinal （2years）	9.73 years n=343	FMS	ROCF	Language 、Math	Standardized Academic Achievement Tests	FMS are significantly and positively correlated with Math(r=0.46,P < 0.05） and Language scores （r=0.46,P < 0.05）.	87.5% Excellent
[82]	Hopkins, 2019	Australia	Cross-sectiona l	7.90 years n=222	FMS	VMI	Reading、Math	Standardized Academic Achievement Tests	FMS are significantly and positively correlated with Math scores(r=0.31**） and Reading scores(r=0.29,P < 0.05）.	87.5% Excellent
[83]	Pienaar, 2013	South Africa	Cross-sectional	6.78 years n=812	FMS	VMI-4	Math、Reading、Writing、and total score	Learning Questionnaire	FMS(VMI) were significantly and positively correlated with Math, Reading and Writing scores and total subject scores(P<0.001) FMS(fine motor coordination) was not significantly related to the math score, but was related to reading, writing and the clustered score for academic performance.	87.5% Excellent

[108]	Kim, 2014	USA	Cross-sectional	Grade 1 n=63	FMS	Teacher Report Questionnaire	Total scores、Math	Standardized Academic Achievement Tests	FMS are significantly and positively associated with total academic scores (β=0.44,p < .01） and Math scores（β=0.32,p < .05）	87.5% Excellent
[84]	Bellocchi, 2017	France	Longitudinal (16-months)	M=64.2±3.9 months n=36	FMS	Developmental Test of Visual Perception (2nd Edition): Visual motor integration (VMI)	reading	Alouette Test-R (reading fluency and accuracy)	FMS was significantly and positively correlated with reading accuracy(r = 0.456, p =< .01) and no correlation with reading fluency.	50.0% low
[98]	Lachance, 2006	USA	Longitudinal (3 years)	M=-5.72-5.83 ±0.33 years n=249	FMS	Beery-Buktenica Developmental Test of Visual-Motor Integration (4th Ed)	Math、reading	Test of Early Mathematical Ability-2nd Edition (TEMA-2)、Keymath revised, Woodcock Johnson - Revised	Positive associations between visual motor integration (VMI) (T0-T3) and math(T1-T3) (r=0.30-0.44, p=0.05)<br><br>Positive associations between VMI (T0-T3) and reading (T0-T3) (r=0.23-0.43, p=0.05)	75.0% Good
[85]	Schatschneider, 2004	USA	Longitudinal (3 years)	n=540<br>Children in kindergarten (T0), grade 1 (T1) grade 2 (T2)	FMS	Beery Test of Visual Motor Integration	reading	Woodcock–Johnson Psychoeducational Test Battery—Revised (WJ–R); The Test of Word Reading Efficiency	FMS in Kindergarten was significantly and positively correlated with reading at the end of Grade 1 and Grade 2(r=0.26-0.27).	68.8% Good

								(TOWRE		
[86]	Michel, 2020	Germany	longitudinal study (3 years)	4-6 years n = 208	FMS	MABC-2	Math	DIRG (Diagnostisches Inventar zu Rechenfertigkeiten im Grundschulalter	FMS in kindergarten were significantly and positively correlated with Math scores in grade 1 and 2(r=.23,p < .05),	81.3% Excellent
[87]	Gashaj, 2018	Switzerland	longitudinal study (1.5 years)	M=6.45±0.37 years n = 136	FMS	MABC-2	math	standardized subtests	FMS in kindergarten were positively and significantly predictive of basic numerical skills at the beginning of second grade (r=.31， p < .05）	87.5% Excellent
[88]	Pitchford, 2016	UK	Cross-sectional	65-80 months n=62	FMS	BOT-2	Math、reading	Wechsler Individual Achievement Test, Second Edition (WIAT-IIUK	Fine Motor Integration were significantly and positively correlated with Math scores(r = 0.569, p < 0.001) and reading(r =0.377, p = 0.003)<br><br>Fine Motor Precision were significantly and positively correlated with Math scores(r = 0.597, p < 0.001),and No significantly correlation with reading.	81.3% Excellent
[89]	Dinehart, 2013	USA	longitudinal study (2 years)	(T0):M=62.5± 3.6 months, grade 2 (T1) n=3 234	FMS	LAP-D	Math、reading	SAT 10 Standardized assessment	Significant weak positive associations found for FMS (T0) and math (T1) (r=0.21-0.33) and reading (r=0.15-0.30)	93.8% Excellent
[90]	Manfra, 2017	USA	longitudinal study (4 years)	4 years n=1442	FMS	LAP-D	Math、reading	GPA	Significant associations between FMS and reading (r=0.18-0.26, p<0.001),maths (r=0.24-0.27, p<0.001) in Grade 3.	93.8% Excellent
[99]	Doyen,	France	Longitudinal		FMS	Peg-moving	Reading、	standard French	FMS (T0) was significantly and positively associated	87.5%

	2017		(7-months)	(T0): 6 years,<br>Year 1(T1)<br>n=86		task	spelling	test (LMC-R,	with reading comprehension (T1) (r=0.24-0.27, p<0.05); spelling (T1) (r=-0.25-.26 p<0.05) in Grade 1	Excellent
[100]	Santi,<br>2014	USA	Cross-sectional	grades 1<br>n=778	FMS	Beery-Buktenica Developmental Test of Visual-Motor Integration (3rd Edition)	Reading	Formal Reading Inventory	Visual motor integration (VMI) skills are related to reading in both grades 1 and 2(r=0.25-0.30, p<0.0015)	93.8%<br>Excellent
[101]	Memis,<br>2016	Turkey	Cross-sectional	grade 1<br>n=168	FMS	Developmental Visual Perception Test (2nd Edition)	Reading	Informal Reading Inventory	Significant association between FMS and reading (r=-0.4544-0.469, p<0.01)	46.7%<br>Low
[102]	Mayes,<br>2009	USA	Cross-sectional	M=8.6±1.5 years<br>n=214	FMS	Grooved Pegboard Test	Reading、math	Wide Range Achievement Test (3rd Edition)	FMS are not related to math and reading.	87.5%<br>Excellent
[91]	Luo,<br>2007	UK	longitudinal study<br>(2 years)	M=67.07-68.61 months<br>n=10060	FMS	The Early Screening Inventory-Revised	math	The Child Assessment Battery	The construct of fine motor skills in kindergartners significantly predicted first grade mathematics achievement over time(one point advantage in FMS led to 1.68 points advantage in initial score and .09 points in growth rates,p <.01).	81.3%<br>Excellent
[92]	Brock,<br>2018	USA	longitudinal study<br>(2 years)	4.8-6.4 years<br>n=259	FMS	Beery–Buktenica Developmental	Math、reading	Woodcock–Johnson Tests of Achievement	Visuomotor integration in kindergarten alone predicted later reading and math in first grade(fit: χ^2 =8.07, p=.78; root-mean-square error of	87.5%<br>Excellent

						Test of VMI—sixth edition		III—Form B	approximation (RMSEA)=0.00; comparative fit index (CFI)=1.00;Tucker–Lewis index (TLI)=1.03)	
[93]	Carlson, 2013	USA	Cross-sectional	10.59±3.39 years n=97	FMS	Visual-Motor Integration (DTVMI)	Math、reading、writing and language	standardized achievement scores from the Kaufman Test of Educational Achievement (KTEA-II), WJ-III (McGrew & Woodcock, and the Wechsler Individual Achievement Test (WIAT-II;	Fine motor Coordination were significantly and positively correlated with reading, writing, and language scores(r=.21-.26,p < .05), and No correlation with math scores.<br>Visual-spatial were significantly and positively correlated with math, reading, writing, and language scores(r=.26-.44,p < .05),	93.8% Excellent
[96]	Mohamed, 2021	Singapore	longitudinal study (2 years)	M=6.78 n=883	FMS	Brigance Inventory of Early Development—3rd Edition (IED-III Standardised	reading、spelling	Wide Range Abilities Test, 4th Edition (WRAT-4)、	FMS were significantly and positively correlated with reading and spelling（r=.06-.42,p < .05)	75.0% Good
[94]	Nesbitt, 2018	USA	longitudinal study (30.8 months)	54.5±3.6 months n = 1138	FMS	Copy Design task	Math	Woodcock Johnson III Achievement Battery (WJ-III）	Kindergarten's visual motor integration were significantly and positively correlated with first grade's math (r = .61)	81.3% Excellent

[103]	Roebers, 2013	Switzerland	Longitudinal (2 years)	M=69.4±4.28 months n=169 Pre-kindergarten (T0): Grade 1 (T2)	FMS	M-ABC-2	math 、reading、spelling (total scores)	standardized tests.	Significant weak positive associations between FMS T1 and Academic achievement total scores (T2)(r=.35,p < .001)	93.8% Excellent
[104]	Julius, 2016	Israel	Longitudinal Study （2 years）	5-8 years n=56	FMS	Grapho-motor task	writing 、reading	Hebrew Handwriting Evaluation (HHE)	FMS was associated with contemporaneous handwriting-legibility and reading.	81.3% Excellent
[53]	Beck, 2016	Denmark	RCT （8 weeks）	7.5 years n=165 ① intervention Group N=**53** ② intervention Group N=**55** ③control group N=**57**	6 weeks 3×60 min	Standardized, diagnostic test	Math	①received mathematical teaching enriched with fine motor activity, ②received mathematical teaching enriched with gross motor activity, ③received non-motor enriched conventional mathematical teaching	②>①>③	75.0% Good
[68]	Damsgaard, 2020	Denmark	RCT	7.61 years n=127 ①	10 min	Recognize the letters “b” and “d”	reading	“b”/“d” training sessions: ①received	①>②③	50.0% low

				intervention Group N=40<br>② intervention Group N=44<br>③control group N=43				teaching enriched with fine motor-enriched<br>②received teaching enriched with gross motor-enriched,<br>③received non-motor teaching		
[54]	Ericsson, 2008	Sweden	RCT<br>（2 years）	Grade 1-3<br>n=251<br>①② intervention Group N=152<br>③control group N=99	2 years<br>5×60 min/week	National tests	Swedish and mathematics	Intervention group ① ② :five lessons per week and one extra lesson of motor training.<br>③ordinary PE for two lessons per week.	①②>③	56.3%<br>Good
[55]	Macdonald, 2021	Australia	Quasi -experimental	m = 6.77±0.40 years<br>n=55<br>① intervention Group N= 19<br>② intervention Group N=19	12 weeks<br>3-4 x 25-30 min/week		Math reading	Intervention group①:Gross motor circuit (4x15 min/week)<br>+Gross motor activities integrated into reading lessons (4x10 min/week)<br>Intervention	Math:①>②>③<br>reading:②>①>③	87.5%<br>Excellent

				③control group N=17				group②:Gross motor circuit (4x15 min/week) ＋Gross motor activities integrated into mathematics lessons (3x15 min/week)<br><br>control group ③:Regular Year 1 English, mathematics and PE program		
[95]	Atsushi, 2017	Japan	RCT pre-post intervention study	M = 85.79 months, n=80 ① intervention Group N= 43 ②control group N=37	3 weeks 3×10 min	Wechsler Intelligence Scale for Children-III, Japanese version (WISC-III;	Math	Intervention group①:fine motor skills training was conducted instead of the reading activity for 3 weeks<br><br>control group ②:Regular reading activity for those same 3 weeks.	①＞②	62.5% Good

[66]	Van den Berg, 2019	Netherlands	Randomized Controlled Trial<br><br>cluster-randomized controlled trial	10.4 years<br>n=369<br>① intervention group<br>②control group=153	5 weeks，4×5-8 min/week	Multiplication Memorization Performance	Math	①: GMS whilst practicing multiplication tables；<br>②:practiced multiplication tables while sedentary	The intervention did not improve, but neither deteriorated children's math performance<br>①=②	93.8%<br>Excellent
[69]	Botha, 2020	South Africa	quasi-experimental research design<br>(Pre-post-tested<br>)	6-7 years<br>n=100<br>① intervention group N=50<br>②control group N=50	12 weeks, 2×30 min/week	ESSI: standardized South African reading and spelling tests	reading and spelling	①：Every session consisted of a 7-min gross motor movements exercise, followed by an 8-min warm-up, four activities (each consisting of 10 min), and a 5-min cool down.<br>②：regular sedentary classroom lessons.	①＞②<br>motor intervention was effective in significantly improving reading and spelling ($p<0.01$)	68.8%<br>Good
[77]	Ericsson, 2012	Sweden	9 years intervention	7–9 years<br>n=220<br>① intervention Group N = 129,	9 years<br>5×45 min/week,<br>and one	Qualified for upper secondary school、sum of marks	language, English, and Mathematics	①Swedish school curriculum, one extra lesson (60 min) of adapted motor training	Boys:①＞②<br>The sum of evaluated marks was higher among boys in the intervention group than in the control group ($P < 0.05$).	87.5%<br>Excellent

				②control group N= 91	extra motor Training lesson (60 min)	in Swedish, Mathematics, English,		②the Swedish standard PE lessons (2×45 = 90 min) of PE per week.	There was a larger proportion of pupils in the intervention than in the control group (96% vs 89%, P < 0.05) who qualified for upper secondary school,	
[56]	Nobre, 2022	Brazil	Randomized Controlled Trial	7-10 years n=280 ① intervention Group N = 140, ②control group N= 140	12-week 3 ×140 min	School Performance Test	reading, writing, math	①With 60 min of academic activities, 20 min recess and snacks, and 60 min of motor practices. ②Participated in a state-sponsored program that provided recreational sports, arts, academic reinforcement (reading, writing, math), and crafts that follow the scholar curriculum.	①>② The intervention group showed significant improvement from pre- to post-test, and higher motor, academic (reading, math) than the comparison group at pos-test (p from 0.031 to <0.001).	87.5% Excellent
[106]	Taverna, 2020	Italy		6 years n=13	10 weeks 2×45 min -1h	Name writing test	writing	Educational activities enhancing fine motor skills and mastering	+ Findings reveal that educational activities impacted positively on children's visual motor coordination component of writing improving VMI scores.	93.8% Excellent

								eye-hand coordination abilities	— No statistically significant difference was detected across the three time points on students' manual dexterity skills.	
[57]	Mullender-Wijnsm, 2016	Netherlands	A Cluster Randomized Controlled Trial(2 school years)	8.1 years ① intervention Group N=249 ②control group N= 250	44 weeks 3×20-30 min.	Child academic monitoring system (CAMS)、One-Minute Test; The Speed Test–Arithmetic	Language, math	①Physically active math and language lessons：In each lesson, 10 to 15 min gross motor skills lesson were spent on math and language activities. The main focus was on constant practice and repetition. For example, the children jumped on the spot 8 times to solve the multiplication sum "2 × 4." ②Regular sedentary classroom lessons.	Math：①>② children in the intervention group had significantly greater gains in mathematics speed test (P < .001; effect size [ES] 0.51), general mathematics (P < .001; ES 0.42), and spelling (P < .001; ES 0.45) scores. No differences were found on the reading test.	87.5% Excellent

[105]	Uhrich, 2007	USA	RCT （6 weeks）	10~11 years n=41 ① intervention Group N=20 ②control group =21	6 weeks 3×20 min	Gates-MacGinitie Reading Test Fourth Edition (GMRT-4)	Reading	Intervention group①:fine motor skills training Ccontrol group ②:snack time	Decoding:①=② Comprehension:①＞②	68.8% Good

Table S2. Results of study quality evaluation using the adapted McMaster Critical Review Form for Quantitative Studies.

References	Items																Score	Quality
	Q1	Q2	Q3	Q4	Q5	Q6	Q7	Q8	Q9	Q10	Q11	Q12	Q13	Q14	Q15	Q16		
[10] Pagani,2010	1	1	1	1	0	1	1	1	1	0	1	1	1	1	1	1	14	87.5% Excellent
[16] Oberer,2018	1	1	1	1	0	1	1	1	1	0	1	1	1	1	1	1	14	87.5% Excellent
[19] Pacheco,2016	1	1	1	1	0	0	1	1	1	1	1	1	1	1	1	1	14	87.5% Excellent
[20] Kim,2017	1	1	1	1	0	1	0	1	1	0	1	1	1	1	1	1	13	81.3% Excellent
[22] Syväoja.2021	1	1	1	1	0	1	0	1	1	1	1	0	1	1	1	1	13	81.3% Excellent
[23] Bruijn, 2019	1	1	1	1	1	1	1	1	1	1	1	0	1	1	1	1	15	93.8% Excellent
[24] Schmidt, 2017	1	1	1	1	0	1	0	1	1	1	1	1	0	1	1	1	13	81.3% Excellent
[25] Lê,2021	1	1	1	1	0	1	0	1	1	0	1	1	1	1	1	1	13	81.3% Excellent

[26] Coetzee,2020	1	1	1	1	0	1	0	1	1	0	1	1	1	1	1	1	13	81.3%<br>Excellent
[28] Milne,2018	1	1	1	1	0	1	1	1	1	1	1	1	1	1	1	1	15	93.8%<br>Excellent
[29] Costa,2018	1	1	1	1	0	1	1	0	1	1	1	1	1	1	1	1	14	87.5%<br>Excellent
[30] Chagas, 2016	1	1	1	0	0	0	0	0	0	0	0	1	1	1	1	1	8	50%<br>Low
[43] Jaakkola, 2015	1	1	1	1	0	1	1	0	1	1	1	1	1	1	1	1	14	87.5%<br>Excellent
[44] Aadland, 2017	1	1	1	1	0	1	0	1	1	1	1	1	1	1	1	1	14	87.5%<br>Excellent
[70] Syväoja,2017	1	1	1	1	0	1	0	1	1	1	0	1	1	1	1	1	13	81.3%<br>Excellent
[71] Batez,2021	1	1	1	1	0	1	1	1	1	1	1	1	1	1	1	1	15	93.8%<br>Excellent
[58] Waal,2020	1	1	1	1	0	1	1	1	1	1	1	0	1	1	1	1	14	87.5%<br>Excellent
[45] Syvaoja 2019	1	1	1	1	0	1	1	0	1	1	1	1	1	1	1	1	14	87.5%<br>Excellent
[46] Aadland,2017	1	1	1	1	0	1	0	1	1	0	1	1	1	1	1	1	13	81.3%<br>Excellent
[47] Lonneman,2011	1	1	1	1	0	1	0	1	0	1	0	1	1	1	1	1	12	75.0%<br>Good
[72] Lima,2020	1	1	1	1	0	0	0	0	0	1	1	1	1	1	1	1	11	68.8%<br>Good
[73] Lopes,2013	1	1	1	1	0	1	1	1	1	1	1	1	1	1	1	1	15	93.8%<br>Excellent
[74] Fernandez-Sanchez,2022	1	1	1	1	0	1	0	0	1	1	0	0	1	1	1	1	11	68.8%<br>Good
[48] Geertsen,2016	1	1	1	1	0	1	1	1	1	1	1	1	0	1	1	1	14	87.5%<br>Excellent
[49] Haapala,2014	1	1	1	1	0	1	1	1	1	1	1	1	1	1	1	1	15	93.8%<br>Excellent
[59] Macdonald,2020	1	1	1	1	1	0	1	1	0	1	1	1	1	1	1	1	14	87.5%<br>Excellent
[50] Rigoli,2012	1	1	1	1	0	0	1	1	1	1	1	1	1	1	1	1	14	87.5%<br>Excellent
[60] Niekerk,2015	1	1	1	1	0	0	0	0	0	0	1	1	0	1	0	1	8	50.0%<br>Low

[75] Katagiri,2021	1	1	1	1	0	0	1	1	1	1	1	1	1	1	1	1	14	87.5% Excellent
[61] Fernández-Méndez.2020	1	1	1	1	0	1	1	0	1	1	0	1	1	1	1	1	13	81.3% Excellent
[78] Ricciardi ,2021	1	1	1	1	0	1	1	1	1	0	1	1	1	1	1	1	14	87.5% Excellent
[62] Gandhi,2012	1	1	1	1	0	1	1	1	1	1	0	1	1	1	1	1	14	87.5% Excellent
[51] Kurdek ,2001	1	1	1	0	1	0	1	1	0	1	1	0	1	1	1	1	12	75.0% Good
[67] Papadimitiriou,2014	1	1	1	1	0	1	1	0	1	0	1	0	1	1	1	1	12	75.0% Good
[52] Son and Meisels,2006	1	1	1	1	0	1	1	1	1	1	1	1	1	1	1	1	15	93.8% Excellent
[63] Giles,2018	1	1	1	1	0	1	0	1	0	1	0	1	1	1	1	1	12	75.0% Good
[64] Escolano-Pérez,2020	1	1	1	1	0	1	1	1	1	1	1	1	1	1	1	1	15	93.8% Excellent
[65] Grissmer,2010	1	1	1	1	0	1	0	0	1	1	0	0	1	1	1	1	11	68.8% Good
[76] Ryu,2021	1	1	1	1	0	1	0	0	1	1	0	1	1	1	1	1	12	75.0% Good
[107] Fernandes,2016	1	1	1	1	0	1	0	1	1	0	1	1	1	1	1	1	13	81.3% Excellent
[79] Duran,2018	1	1	1	1	0	1	1	1	1	1	1	1	1	1	1	1	15	93.8% Excellent
[97] Suggate,2019	1	1	1	1	0	1	0	0	1	1	1	0	1	1	1	1	12	75.0% Good
[80] Sortor,2003	1	1	1	1	0	1	0	0	1	1	0	0	1	1	1	1	11	68.8% Good
[81] Sulik,2018	1	1	1	1	0	1	1	1	1	0	1	1	1	1	1	1	14	87.5% Excellent
[82] Hopkins,2019	1	1	1	1	0	1	1	1	1	0	1	1	1	1	1	1	14	87.5% Excellent
[83] Pienaar,2013	1	1	1	1	0	1	1	1	1	0	1	1	1	1	1	1	14	87.5% Excellent
[108] Kim,2014	1	1	1	1	0	1	1	1	1	0	1	1	1	1	1	1	14	87.5% Excellent
[84] Bellocchi,2017	1	1	1	1	0	0	0	0	0	0	0	0	1	1	1	1	8	50.0% low

[98] Lachance,2006	1	1	1	1	0	1	0	0	1	1	1	0	1	1	1	1	12	75.0%<br>Good
[85] Schatschneider,2004	1	1	1	1	0	1	0	0	1	1	0	0	1	1	1	1	11	68.8%<br>Good
[86] Michel,2020	1	1	1	1	0	1	0	1	1	0	1	1	1	1	1	1	13	81.3%<br>Excellent
[87] Gashaj,2018	1	1	1	1	0	1	1	1	1	0	1	1	1	1	1	1	14	87.5%<br>Excellent
[88] Pitchford,2016	1	1	1	1	0	1	0	1	1	0	1	1	1	1	1	1	13	81.3%<br>Excellent
[89] Dinehart,2013	1	1	1	1	0	1	1	1	1	1	1	1	1	1	1	1	15	93.8%<br>Excellent
[90] Manfra,2017	1	1	1	1	0	1	1	1	1	1	1	1	1	1	1	1	15	93.8%<br>Excellent
[99] Doyen,2017	1	1	1	1	0	1	1	1	1	0	1	1	1	1	1	1	14	87.5%<br>Excellent
[100] Santi,2014	1	1	1	1	0	1	1	1	1	1	1	1	1	1	1	1	15	93.8%<br>Excellent
[101] Memis, 2016	1	1	1	1	0	0	0	0	0	0	0	0	0	1	1	1	7	43.8%<br>Low
[102] Mayes,2009	1	1	1	1	0	1	1	1	1	0	1	1	1	1	1	1	14	87.5%<br>Excellent
[91] Luo,2007	1	1	1	1	0	1	0	1	1	0	1	1	1	1	1	1	13	81.3%<br>Excellent
[92] Brock,2018	1	1	1	1	0	1	1	1	1	0	1	1	1	1	1	1	14	87.5%<br>Excellent
[93] Carlson,2013	1	1	1	1	0	1	1	1	1	1	1	1	1	1	1	1	15	93.8%<br>Excellent
[96] Mohamed,2021	1	1	1	1	0	1	0	1	0	0	1	1	1	1	1	1	12	75.0%<br>Good
[94] Nesbitt,2018	1	1	1	1	0	1	0	1	1	0	1	1	1	1	1	1	13	81.3%<br>Excellent
[103] Roebers,2013	1	1	1	1	0	1	1	1	1	1	1	1	1	1	1	1	15	93.8%<br>Excellen
[104] Julius,2016	1	1	1	1	0	1	0	1	1	0	1	1	1	1	1	1	13	81.3%<br>Excellent
[53] Beck,2016	1	1	1	1	0	1	0	0	1	0	1	1	1	1	1	1	12	75.0%<br>Good
[68] Damsgaard,2020	1	1	1	1	0	0	0	0	0	0	0	0	1	1	1	1	8	50.0%<br>low

[54] Ericsson,2008	1	1	1	1	0	1	0	0	0	0	0	0	1	1	1	1	9	56.3%<br>Good
[55] Macdonald,2021	1	1	1	1	0	1	1	1	1	0	1	1	1	1	1	1	14	87.5%<br>Excellent
[95] Atsushi,2017	1	1	1	1	0	1	0	0	0	0	1	1	0	1	1	1	10	62.5%<br>Good
[66] Berg,2019	1	1	1	1	0	1	1	1	1	1	1	1	1	1	1	1	15	93.8%<br>Excellent
[69] Botha,2020	1	1	1	1	0	1	0	0	0	0	1	1	1	1	1	1	11	68.8%<br>Good
[77] Ericsson,2012	1	1	1	1	0	1	1	1	1	0	1	1	1	1	1	1	14	87.5%<br>Excellent
[56] Nobre,2022	1	1	1	1	0	1	1	1	1	0	1	1	1	1	1	1	14	87.5%<br>Excellent
[106] Taverna,2020	1	1	1	1	0	1	1	1	1	1	1	1	1	1	1	1	15	93.8%<br>Excellent
[57] Mullender-Wijnsma,2016	1	1	1	1	0	1	1	1	1	0	1	1	1	1	1	1	14	87.5%<br>Excellent
[105] Uhrich,2007	1	1	1	1	0	1	0	0	1	0	1	0	1	1	1	1	11	68.8%<br>Good

Note. Q1: Was the study purpose stated clearly?; Q2: Was the relevant background literature reviewed?; Q3: Was the design appropriate for the research question?; Q4: Was the sample described in detail?; Q5: Was the sample size justified?; Q6: Was informed consent obtained?; Q7: Were the outcome measures reliable?; Q8: Were the outcome measures valid?; Q9: Was the method described in detail; Q10: Were results reported in terms of statistical significance?; Q11: Were the analysis methods appropriate?; Q12: Was importance for the practice reported?; Q13: Were any drop-outs reported?; Q14: Were the conclusions appropriate given the study methods?; Q15: Are there any implications for practice given the results of the study?; Q16: Were limitations of the study acknowledged and described by the authors?

1: Yes, 0: No.
